# Evidence for in-gap surface states on the single phase SmB_6_(001) surface

**DOI:** 10.1038/s41598-017-12887-2

**Published:** 2017-10-09

**Authors:** Toshio Miyamachi, Shigemasa Suga, Martin Ellguth, Christian Tusche, Claus M. Schneider, Fumitoshi Iga, Fumio Komori

**Affiliations:** 1 The Institute for Solid State Physics, The University of Tokyo, Kashiwa, Chiba 277-8581 Japan; 2 Institute of Scientific and Industrial Research, Osaka University, Ibaraki, Osaka 567-0047 Japan; 3 Peter Grünberg Institut (PGI-6), Forschungszentrum Jülich, 52425 Jülich, Germany; 4 Institut für Physik, Johannes-Gutenberg-University, Mainz, 55128 Mainz Germany; 5 Fakultät für Physik, Universität Duisburg-Essen, 47057 Duisburg, Germany; 6 College of Science, Ibaraki University, Mito, Ibaraki 310-0056 Japan

## Abstract

Structural and electronic properties of the SmB_6_(001) single-crystal surface prepared by Ar^+^ ion sputtering and controlled annealing are investigated by scanning tunneling microscopy. In contrast to the cases of cleaved surfaces, we observe a single phase surface with a non-reconstructed *p*(1 × 1) lattice on the entire surface at an optimized annealing temperature. The surface is identified as Sm-terminated on the basis of spectroscopic measurements. On a structurally uniform surface, the emergence of the in-gap state, a robust surface state against structural variation, is further confirmed inside a Kondo hybridization gap at 4.4 K by temperature and atomically-resolved spatial dependences of the differential conductance spectrum near the Fermi energy.

## Introduction

The Kondo insulator samarium hexaboride (SmB_6_) has attracted great attention over decades due to its strong electron correlation. It exhibits a metal-insulator transition with decreasing temperature as a consequence of a gap opening triggered by the hybridization of localized 4f states with itinerant 5d states^1^. A particular interest is directed to the plateau in the electrical resistivity in the low temperature regime (< 5 K)^2^. An in-gap state inside the Kondo hybridization gap was proposed as a possible origin of this resistance plateau^3^. Theoretical predictions that SmB_6_ is a promising candidate for a topological Kondo insulator with a topologically protected surface state^4,5^ revive the intense interest in experimental studies to identify the origin of the in-gap state^6,7^. However, spatially-resolved surface characterizations by scanning tunneling microscopy (STM) have revealed that several types of surface structures randomly coexist on the cleaved SmB_6_(001) surfaces^8–12^. This hampers the investigation of its intrinsic surface electronic properties by spatially-integrated methods. Furthermore, the cleavage of SmB_6_ results in non-reproducible relative weight of the surface structures, prohibiting reliable discussion on electronic as well as atomic structures of the prepared surface. Thus, the establishment of a new reproducible preparation method of the single phase SmB_6_ surface at least over sub-μm^2^ is mandatory not only from the viewpoint of the fundamental material science but also from the application point of view. As an alternative to the crystal cleavage, a cyclic Ar^+^ ion sputtering and subsequent annealing is recently proposed to reproducibly prepare a single-phase SmB_6_(001) surface by spatially-integrated observations with low energy electron diffraction (LEED)^13^. Nevertheless, the atomic structure of such a surface still remains to be confirmed for detailed discussion of its in-gap surface state because a non-reconstructed *p*(1 × 1) LEED pattern could appear for disordered SmB_6_(001) surface^14^. Furthermore, previous LEED studies have reported the existence of superstructures even on the surface prepared by this method^15,16^, which implies the difficulty of controlling appropriate preparation conditions.

In this study, we report real-space STM characterizations of a single-phase SmB_6_(001) surface prepared by a cyclic Ar^+^ ion sputtering and optimally controlled annealing on the atomic scale with a reliable evaluation of its in-gap state. We find that the surface quality is gradually improved when increasing annealing temperature up to 1030 °C. Atomically-resolved STM observations combined with spectroscopic measurements have revealed that at an optimized annealing temperature the entire surface is composed of a non-reconstructed *p*(1 × 1) lattice with Sm-termination. Furthermore, temperature and atomically-resolved spatial dependences of the differential conductance spectrum near the Fermi energy identify the in-gap state with a characteristic peak-dip feature emerging inside the Kondo hybridization gap at 4.4 K as a robust surface state against structural variation.

## Results

**Figure 1:**
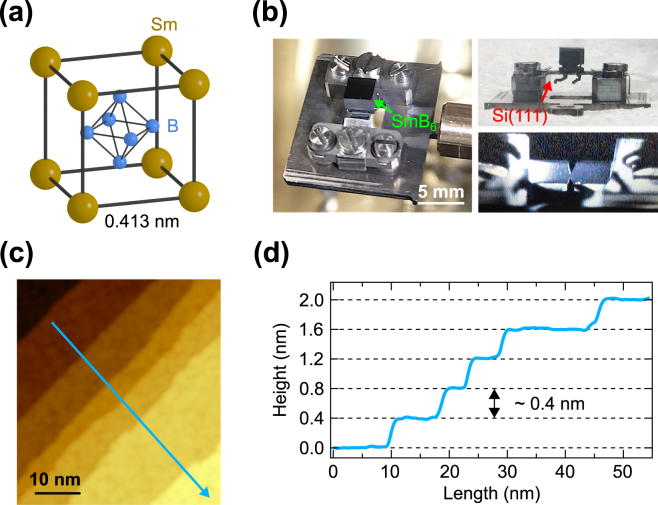
(**a**) Schematic crystal structure of SmB_6_. (**b**) Photographs of the SmB_6_(001) single crystal and its mounting. (**c**) STM image of the SmB_6_(001) surface annealed at 1030 °C. (**d**) STM height profile of atomic steps along the blue line in (**c**). The interval between the grid lines in height is nearly equal to the lattice constant of SmB_6_.

The crystal structure of SmB_6_ is schematically displayed in Fig. 1(a). In the CsCl-type structure, one Sm atom and one B_6_ octahedron occupy the cubic lattice with the lattice constant of 0.413 nm. Figure 1(b) displays photographs of the SmB_6_(001) single crystal in this study, which is fixed to a Si(111) substrate and mounted to the STM sample holder. Note that the single crystal is annealed via direct current heating of the Si(111) substrate. The flatness of the surface can be recognized from a mirror image of the STM tip approaching from the bottom. Atomic steps are indeed visible in an STM image of the SmB_6_(001) surface prepared by Ar^+^ ion sputtering and subsequent annealing at 1030 °C as shown in Fig. 1(c). The corresponding STM height profile shown in Fig. 1(d) clearly gives the step height of ~ 0.4 nm nearly identical to the lattice constant of the SmB_6_(001) plane.

**Figure 2:**
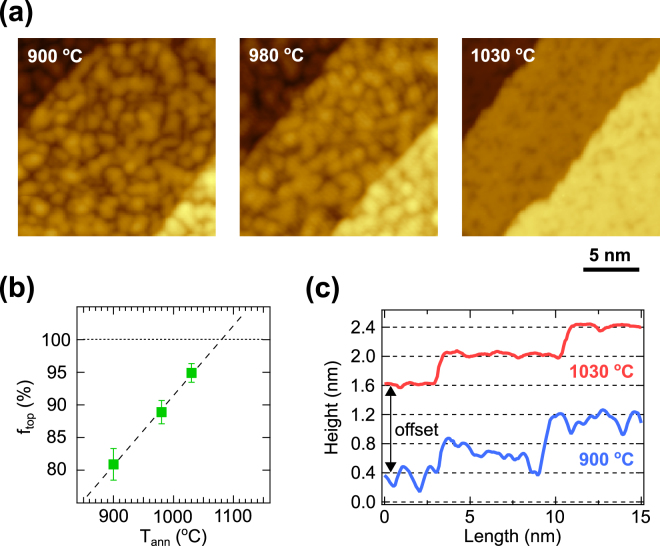
(**a**) STM images of the SmB_6_(001) surface annealed at 900, 980 and 1030 °C. (**b**) *f*_*top*_ values as a function of the annealing temperature with the least squares fit. (**c**) Typical STM height profiles of the SmB_6_(001) surface annealed at 900 and 1030 °C. The interval between the grid lines in height is nearly equal to the lattice constant of SmB_6_.

We first investigate the annealing temperature dependence of the SmB_6_(001) surface. A series of the SmB_6_(001) surfaces annealed at 900, 980 and 1030 °C are displayed in Fig. 2(a). While terraces are separated by monatomic steps for all the annealing temperatures, we find clear differences in the density of defects on the surface. At 900 °C, the surface is composed of small islands with a high density of defects. At higher temperature annealing, the coalescence of the islands is promoted, resulting in the reduction of the defect density. The surface quality is considerably improved after 1030 °C annealing. Since Ar^+^ ion sputtering increases the concentration of Sm atoms near the surface^13^, the annealing process reduces the defect density and flattens the surface with a Sm-termination. The fraction of the topmost layer, *f*_*top*_, as a function of the annealing temperature is plotted in Fig. 2(b). An extrapolation of a monotonous increase of *f*_*top*_ with the annealing temperature gives the maximum *f*_*top*_ at 1080 °C, in line with the evaluation of the quality of the Sm-terminated surface by a Sm/B Auger peak ratio^13^.

To get further insights into the SmB_6_(001) surface, we focus on the defect depth. Figure 2(c) displays typical STM line profiles on the surface annealed at 900 and 1030 °C. After annealing at 900 °C, a wide range of the depth distribution of more than one lattice constant (~ 0.4 nm) is observed, strongly implying the presence of several types of surface terminations. After annealing at 1030 °C, however, the defects show only one level of ~ 80 pm in depth, corresponding well to the height difference between the Sm plane and the underlying topmost B atom in the B_6_ octahedron on the Sm-terminated surface as reported for cleaved samples^10^ [see also Fig. 1(a)]. Taking the lower vaporization energy of Sm relative to B into consideration^17^, annealing at 1030 °C or up to 1080 °C improves the surface quality by filling the defect sites with the Sm atoms.

**Figure 3:**
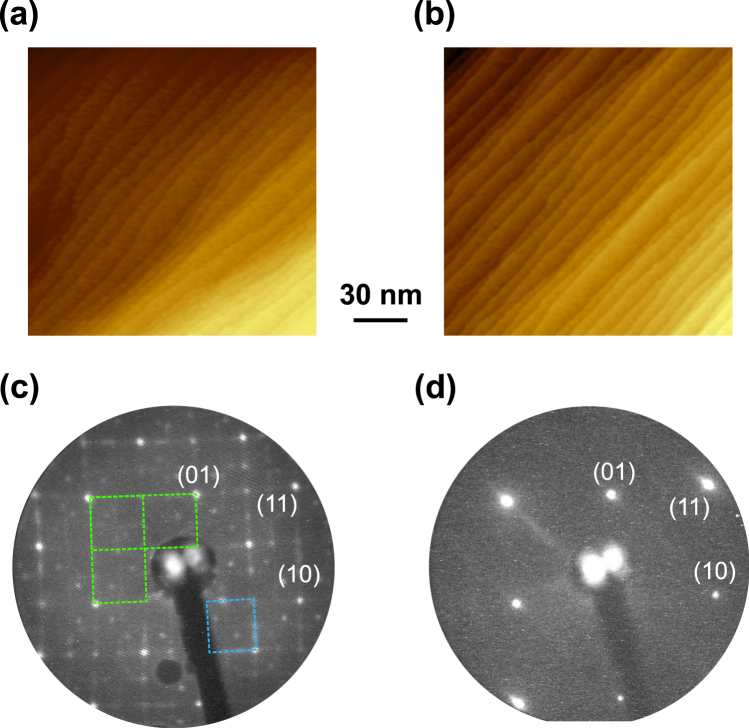
Large scale STM images of the SmB_6_(001) surface annealed at (**a**) 900 and (**b**) 1030 °C. (**c**) LEED pattern of the sample annealed at 900 °C. Superstructure spots of *c*(2 × 2) and (1 × 2) or (2 × 1) are indicated by blue and green rectangles. (**d**) LEED pattern of the sample annealed at 1030 °C. LEED patterns are taken at the electron energy of 38 eV.

We have further confirmed that the observed annealing temperature dependence is consistently reflected in a macroscopic scale. Figure 3(a) and (b) display large scale STM images of the SmB_6_(001) surface annealed at 900 and 1030 °C. Both surfaces exhibit similar vicinal structures with a high density of atomic steps, which arise from the slight miscut angle of the crystal from the [001] direction. However, the LEED pattern of the surface changed drastically with increasing annealing temperature as compared between Fig. 3(c) and (d). The complex LEED pattern of the sample annealed at 900 °C strongly indicates the coexistence of several types of reconstructed surfaces, e.g., *c*(2 × 2), (1 × 2) or (2 × 1) reconstructions, in line with the interpretation of the STM observations in Fig. 2. In contrast, the LEED pattern after 1030 °C annealing shows a *p*(1 × 1) structure with no additional superstructure spots in the measured energy range between 15 and 120 eV. The faint streaks appearing in the LEED pattern at this annealing temperature reflect the alignment of the steps in one direction [see also Fig. 3(a) and (b)]. Still no obvious difference in the terrace width is observed between the surfaces annealed at 900 and 1030 °C. Thus, the role of high-density steps acting as strain relievers to stabilize the surface lattice^18^ is minor, and the annealing at an optimized temperature is essential for the formation of the *p*(1 × 1) lattice. Note that each LEED pattern is measured immediately after the STM observations, and thus a degradation of the surface can be excluded as a possible origin of the change in the LEED pattern^14^.

The annealing temperature dependence investigated by STM and LEED reveals that the surface quality improves gradually when raising annealing temperature, and the single phase SmB_6_(001) surface with a non-reconstructed *p*(1 × 1) lattice emerges on the entire surface at around 1030 °C. We have then performed atomically-resolved STM observations of the SmB_6_(001) surface to check the surface termination. Atomic-scale ordered structures of the non-reconstructed SmB_6_(001) surface with Sm- and B- terminations have been reported by STM previously on the cleaved surfaces^8–12^. The B-terminated surface derived from B apex atoms in the B_6_ octahedra exhibits a periodicity corresponding to the lattice constant of SmB_6_. On the Sm-terminated surface, however, a lattice with alternating rows of Sm and B atoms with different heights would appear due to the existence of Sm and B sublattices, leading to a periodicity with 1/$\sqrt{2}$ of the SmB_6_ lattice constant^9^.

**Figure 4:**
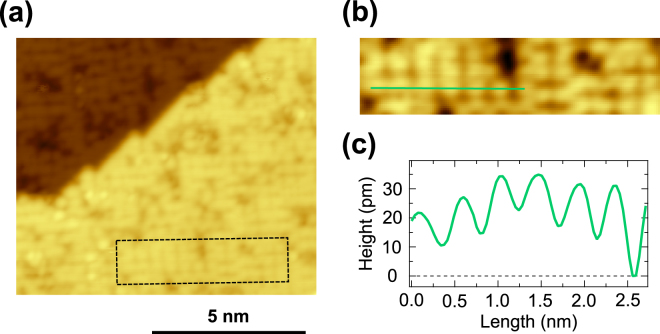
(**a**) Atomically-resolved STM image of the SmB_6_(001) surface annealed at 1030 °C. (**b**) Zoomed STM image from (**a**). The image size corresponds to the rectangle in (**a**). (**c**) STM height profile along the green line in (**b**).

Figure 4(a) displays an atomically-resolved STM image of the SmB_6_(001) surface annealed at 1030 °C. We still observe a non-negligible amount of defects, which induces the local disorder on the surface. A relatively ordered region zoomed from the dashed rectangle region in Fig. 4(a) is displayed in Fig. 4(b), forming the non-reconstructed *p*(1 × 1) lattice as confirmed by LEED. However, the STM height profile shown in Fig. 4(c) reveals that the lateral periodicity of the *p*(1 × 1) lattice distributes between 300 and 500 pm. Furthermore, its height changes non-systematically. Thus, the surface disorder caused by a certain amount of defects makes it difficult to determine the surface termination only by atomically-resolved STM observations. Note that the maximum annealing temperature in this study is restricted up to ~1030 °C since the temperature of the underlying Si(111) substrate nearly reaches its melting point of ~1200 °C^19–21^, although further increasing annealing temperature may decrease the defect density and improve the surface quality.

**Figure 5:**
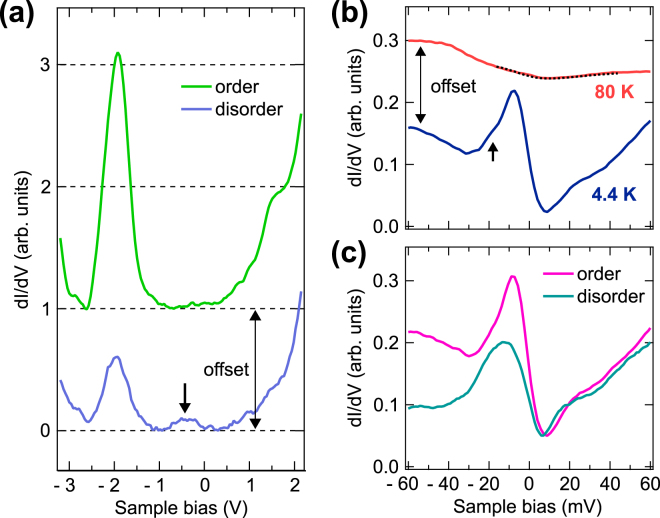
(**a**) dI/dV spectra over a wide range of sample bias from −3.2 V to 2.2 V recorded on ordered (Sm-terminated), and disordered regions at T = 80 K. The arrow indicates the broad feature. (**b**) Temperature dependence of the spatially averaged dI/dV spectra near the Fermi energy. The dotted line on the dI/dV spectrum at T = 80 K is a Fano fit (see text) with a q-factor of 0.56, Γ of 21.6 ± 0.6 meV and E_0_ of ~ 8 meV. The arrow indicates the fine shoulder structure possibly derived from localized bulk f-states. (**c**) Spatial dependence of the dI/dV spectra near the Fermi energy recorded on the ordered region, and the disordered region near defects.

Spectroscopic measurements can also play a crucial role to determine the surface termination. We especially focus on the energy level of the surface state derived from the B 2p dangling bonds. An angle-resolved photoemission (ARPES) study supported by density functional theory revealed that the energy level of the B 2p surface state depends strongly on the surface termination, i.e, it is located near the Fermi energy for the B-terminated surface, while it shifts about 2 eV below the Fermi energy for the Sm-terminated surface^13,22^. Figure 5(a) displays the dI/dV spectra of the SmB_6_(001) surface annealed at 1030 °C for both on ordered regions and on disordered regions near defects. Being independent of the surface quality, a common peak structure located at −2.0 V is observed. Its energy level corresponds nicely to that of the surface state derived from the B 2p dangling bonds on a Sm-terminated surface. Thus, combining with the results of atomically-resolved STM observations shown in Fig. 4, we conclude the Sm-terminated single phase with non-reconstructed *p*(1 × 1) lattice is formed on the SmB_6_(001) surface prepared by Ar^+^ ion sputtering and annealing at an optimized temperature.

The structural information on the SmB_6_(001) surface could be further extracted from the spatial dependence of the B 2p peak intensity. We find that the B 2p peak intensity is weak on disordered regions near defects. In addition, a feature located at around −0.5 V is occasionally observed in the dI/dV spectrum as indicated by an arrow in Fig. 5(a). On the other hand, the feature (−0.5 V) disappears in the dI/dV spectrum with the strong B 2p peak intensity on ordered regions. A similar tendency is reported on a cleaved SmB_6_(001) surface, and is interpreted in terms of the existence of disordered regions with random Sm- and B- terminations^22^. Thus, in accord with atomically-resolved STM observations shown in Fig. 4, the observed spatial dependence of the B 2p peak intensity in the dI/dV spectra in Fig. 5(a) is attributed to the degree of surface disorder caused by defects.

Finally, we discuss the electronic properties of the SmB_6_(001) surface near the Fermi energy. Figure 5(b) displays the temperature dependence of the dI/dV spectrum near the Fermi energy. At 80 K, the dI/dV spectrum exhibits a typical asymmetric lineshape interpreted as the Kondo hybridization gap^6,7,9,12^. The asymmetric gap indicates the Fano resonance described by a quantum interference between tunneling paths into the Kondo resonance and into the conduction-band states^23^. The dI/dV spectrum with such a quantum interference can be expressed by the Fano equation^24^ as

1 $$\frac{dI}{dV}\propto A\cdot \frac{{(q+\varepsilon )}^{2}}{{\mathrm{(1}+\varepsilon )}^{2}}+B,\quad \varepsilon =\frac{eV-{E}_{0}}{\Gamma }$$

where Γ is a half width at half maximum of the size of the hybridization gap, E_0_ is the peak (dip) position of the Kondo resonance with respect to the Fermi energy, and q, so-called a q factor, describes the lineshape of the Fano resonance, respectively. The relationship between Γ and the Kondo temperature *T*_*k*_ can be expressed as 2Γ = $\sqrt{{\mathrm{(5.4}{k}_{B}T)}^{2}+{\mathrm{(2}{k}_{B}{T}_{K})}^{2}}$ for finite temperatures *T* ^25^. In this study, the experimental dI/dV spectrum is simulated with a standard Fano model to extract Γ and *T*_*k*_, while the importance of advanced analyses such as a cotunnleing model or the Fano model with the Gaussian peak fitting is proposed to fully describe the dI/dV spectra especially at higher energy ranges in the Kondo lattice system^9,12^. The evaluated Γ of ∼ 21.6 meV from the Fano fit to the dI/dV spectrum recorded at 80 K gives *T*_*k*_ of ~ 150 K [see the dotted line in Fig. 5(b)], in good agreement with the recent ARPES studies^7^. A drastic change occurs in the dI/dV spectrum with decreasing temperature. We find the emergence of the in-gap state with a characteristic peak-dip feature inside the Kondo hybridization gap at 4.4 K. The observed temperature dependence of the dI/dV spectrum is also reported by previous STM studies on the cleaved SmB_6_(001) surfaces^9,12^, thus ensuring intrinsic electronic properties of the SmB_6_(001) surface prepared by Ar^+^ ion sputtering and annealing in this study.

A remaining question is whether the observed in-gap state dominantly reflect surface- or bulk- related characters. We here discuss the robustness of the in-gap state with a pronounced peak structure located at ~ −8 meV against spatial variations. Since surface states are expected to be more sensitive to the surface disorder often induced by defects than bulk states, the comparison of the dI/dV spectra recorded on ordered and disordered regions would provide the additional information on the nature of the in-gap state. Figure 5(c) displays the spatial dependence of the dI/dV spectra near the Fermi energy recorded on ordered and disordered regions of the SmB_6_(001) surface at 4.4 K. The pronounced peak structure observed on the ordered region is noticeably suppressed on the disordered region near defects.

According to the atomic-scale spatial dependence measurements of the dI/dV spectra (Supplementary Fig. S1), the peak intensity is significantly reduced near defects. However, even atop the defect, the peak structure still remains and the intensity is minimum. The peak intensity significantly recovers at the ordered site even 0.5 nm apart from the defect as shown in Fig. S1(c). On the ordered region, the peak intensity exhibits no essential site dependence. These results reveal that the in-gap state with the pronounced peak structure is sensitive to the structural variation on the surface, but survives on the whole surface irrespective of defects. The observed structural robustness of the in-gap state would be related to the topological protection that is one of the main characteristics as a topological surface state. Note that, as a consequence of the reduction of the peak intensity located at −8 meV near the defect, the fine shoulder structure indicated by the arrow in Fig. 5(b) is clearly recognized as a peak structure at −15 meV [See the dotted line in Supplementary Fig. S1(d)]. Its energy position and intensity are almost independent of the structural variation on the surface. Thus, in contrast to the surface-state-derived peak structure located at −8 meV, the localized bulk f-states could dominantly contribute to this peak structure as recently reported for the cleaved SmB_6_(001) surface by STM measurements at lower temperatures with a lower bias-voltage modulation^12^.

In summary, real-space surface characterizations by STM successfully prove the formation of the Sm-terminated single-phase SmB_6_(001) surface with a non-reconstructed *p*(1 × 1) lattice by Ar^+^ ion sputtering and controlled annealing. Temperature and spatial dependent spectroscopic measurements near the Fermi energy indicated that the in-gap state emerging inside the Kondo hybridization gap at 4.4 K is a robust surface state against structural variations, which could reflect the intrinsic electronic properties of SmB_6_ potentially as a topological Kondo insulator. The validity of Ar^+^ ion sputtering and controlled annealing to reproducibly achieve the single-phase SmB_6_(001) surface demonstrated here allows the access for intrinsic electronic properties of SmB_6_ by spatially-integrated techniques towards application purposes as well as further fundamental researches in material science.

## Methods

### SmB_6_(001) single crystal

In this study, the same SmB_6_(001) single crystal as in ref.^13^ was used, which had been grown by the floating-zone melting method^26^, then cut and polished along the (001) plane determined by Laue x-ray diffraction. The surface was prepared *in situ* by repeated cycles of Ar^+^ ion sputtering with 1.0 keV energy and subsequent controlled annealing. The annealing temperature was measured on the SmB_6_(001) crystal using a pyrometer.

### Measurement conditions

STM measurements were performed in ultrahigh vacuum (< 4.0 × 10^−11^ mbar) at 4.4 and 80 K by using electrochemically-etched W tips. The differential conductance spectra, dI/dV, were recorded using a lock-in technique with a bias-voltage modulation of 3 and 20 mV at 722 Hz. Spatially-averaged structural information of the surface was also investigated by LEED at room temperature.

## Electronic supplementary material

Supplementary Information
