# Supplementary material for: Evidence for in-gap surface states on the single phase SmB6(001) surface
Source: Sci Rep. 2017 Oct 9;7:12837. doi: 10.1038/s41598-017-12887-2 (PMC5634509; doi:10.1038/s41598-017-12887-2)
Supplement: Supplementary file 1 — Supplementary Information [file 41598_2017_12887_MOESM1_ESM.pdf]

Supplementary Information for Evidence for in-gap surface states on the single phase SmB_6(001) surface

Toshio Miyamachi[1], Shigemasa Suga[2,3], Martin Ellguth[4], Christian Tusche[3,5], Claus M. Schneider[3,5], Fumitoshi Iga[6], and Fumio Komori[1]

[1]The Institute for Solid State Physics, The University of Tokyo, Kashiwa, Chiba 277-8581, Japan

[2]Institute of Scientific and Industrial Research, Osaka University, Ibaraki, Osaka 567-0047, Japan

[3]Peter Grünberg Institut (PGI-6), Forschungszentrum Jülich, 52425 Jülich, Germany

[4]Institut für Physik, Johannes-Gutenberg-University, Mainz, 55128 Mainz, Germany

[5]Fakultät für Physik, Universität Duisburg-Essen, 47057 Duisburg, Germany

[6]College of Science, Ibaraki University, Mito, Ibaraki 310-0056, Japan

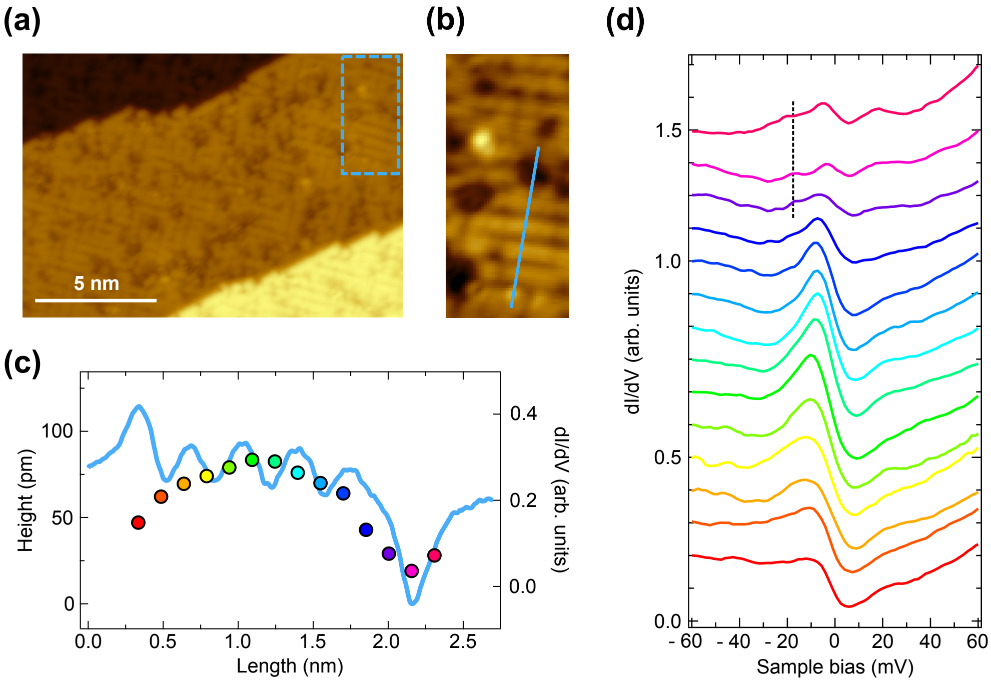

Figure S 1: Spatial dependence of the dI/dV spectra near the Fermi energy recorded at 4.4 K. (a) Atomically-resolved STM image of the $SmB_6(001)$ surface annealed at 1030 °C. (b) Zoomed STM image from (a). The image size corresponds to the rectangle in (a). (c) (Left axis) STM height profile along the blue line in (b). Two types of defects, a small protrusion and a dip, are visible at ~ 0.3 (left) and at 2.2 (right) nm, respectively. (Right axis) dI/dV intensity plot along the blue line in (b). At each position, the magnitude of the dI/dV intensity of the peak structure located at ~ -8 meV in (d) is plotted. (d) A series of 14 dI/dV spectra recorded along the blue line in (b). The colors indicate the recorded positions of the dI/dV spectra marked as dots with the same colors in (c), i.e, the spectra from bottom to top in (d) corresponds to the dots from left to right in (c). The dotted line indicates the energy position of the fine structure located at ~ -15 meV in the in-gap state.
